# Attitudes of nurses toward telenursing and influencing factors in resource-limited settings: Northwest Ethiopia 2022

**DOI:** 10.3389/fdgth.2024.1366242

**Published:** 2025-01-03

**Authors:** Fikadu Wake Butta, Biniyam Chaklu Tilahun, Berhanu Fikadie Endehabtu, Adamu Ambachew Shibabaw, Alex Ayenew Chereka, Ayenew Sisay Gebeyew, Mekides Molla Reda, Gemeda Wakgari Kitil, Teshome Demis Nimani

**Affiliations:** ^1^ Department of Health Informatics, College of Health Sciences, Mattu University, Mattu, Ethiopia; ^2^ Departments of Health Informatics, Institute of Public Health, University of Gondar, Gondar, Ethiopia; ^3^ Departments of Health Informatics, College of Health Sciences, Debre Markos University, Debre Markos, Ethiopia; ^4^ Department of Midwifery, College of Health Sciences, Mattu University, Mattu, Ethiopia; ^5^ Departments of Biostatics and Epidemiology, College of Health Science, Haramaya University, Harar, Ethiopia

**Keywords:** attitude, nurse, Ethiopia, telenursing, telenursing care, Statistical Package for Social Science (SPSS), variance inflation factor (VIF), World Health Organization (WHO)

## Abstract

**Background:**

The worldwide scarcity of nurses is a pressing concern, with the World Health Organization predicting a deficit of 5.9 million nurses globally by 2025. Notably, 89% of this shortage is expected to impact low- and middle-income countries. To address the growing demand for nursing professionals, the concept of telenursing care is being considered. However, there is limited evidence regarding nurses' attitudes towards telenursing care in Ethiopia. This study aims to understand how nurses feel about telenursing care and the factors related to it at a specialized teaching referral hospital in northwest Ethiopia.

**Method:**

We conducted a cross-sectional study at a specialized teaching referral hospital, employing a simple random sampling technique to gather information from 423 nurses. The study took place from July 28 to December 19, 2022/23. Descriptive statistics, including tables and bar graphs, were utilized. Additionally, a binary logistic regression analysis was conducted with 95% confidence intervals and a significance level of *P* < 0.05 to identify factors influencing nurses' attitudes toward telenursing.

**Result:**

Out of the total 416 nurses who responded, representing a response rate of 98.35%, 39.7% exhibited favorable attitudes towards telenursing care. Factors associated with nurses' attitudes included awareness, source of information, social media use, knowledge, computer access, digital training, internet access, and computer training.

**Conclusions:**

The findings indicate a low level of positive attitudes towards telenursing care among nurses. To enhance future acceptance, use, and implementation, policymakers, higher education institutions, and other stakeholders should collaborate to improve nurses' attitudes toward telenursing care, taking into consideration various factors and user preferences.

## 1 Introduction

yIn the present-day context, the demand for nursing services has evolved rapidly and become more complex (1, 2). Globally, among the most pressing challenges for public health systems and the nursing community is the increasing shortage of nurses (3). Many countries today grapple with various health issues, including an aging population, the need for home care, a shortage of nursing staff for direct patient care services at home or remotely, the challenges posed by pandemic diseases like COVID-19, and conflicts (4–7).

Before the outbreak of the coronavirus disease (COVID-19), the global nurse shortage was widely recognized. In 2020, the World Health Organization (WHO) published its first' State of the World's Nursing' report, revealing that 27.9 million nurses were working worldwide. The report predicted a shortage of 5.9 million nurses in low- and middle-income countries, accounting for 89% of these shortages, with notable regional differences (8, 9).

The primary objective of healthcare facilities in Ethiopia is to deliver high-quality healthcare to patients. The quality of nursing care significantly influences overall healthcare quality, especially as nurses play a crucial role in providing direct primary healthcare in rural and remote areas, as well as delivering outstanding care to patients in hospitals (10, 11). So, nurses play crucial roles in managing the quality of treatment and enhancing healthcare outcomes, as they are the most essential component in developing nations for providing high-quality healthcare (10, 12, 13). However, the availability of resources, nursing documentation, technology, and interactions with patients and other healthcare providers all have an impact on the standard of nursing care (10, 13).

Additionally, nurses constitute the largest group of healthcare providers in Ethiopia. As of 2016, there were a total of 50,604 nursing professionals, and it is anticipated that this number will increase to 127,299 professionals by 2025 (11, 14). According to a 2018 World Bank estimate, Ethiopia had a nurse and midwife density of 0.7135 per 100,000 population (15).

Notably, the majority of healthcare facilities in Ethiopia lack a retention plan, leading to significant nurse turnover. Consequently, the nurse-to-patient ratio varies from 1:6 to 1:12, depending on the institution (10). As an essential linkage in the delivery of medical services between patients, doctors, and other nursing medical practitioners, nurses contribute significantly to the preservation of public healthcare because of their high relevance for interventions and treatment in developing countries (7, 16–18).

Nevertheless, a global shortage of nursing practitioners persists in the present day (3, 7). Nurses contribute to a growing field of specialists by incorporating novel technologies into the healthcare procedure (1, 2, 4, 19). The emergence of telehealth nursing is a significant innovation that fundamentally transforms the delivery of nursing healthcare (4, 7, 19–21). To address the expanding need for nursing professionals, the exploration of new technologies, such as telehealth nursing (telemedicine in nursing), is underway as a solution to meet the demands for patient care (22).

Telenursing integrates nursing with computer and information sciences for the handling and exchange of data, information, and knowledge within nursing practice (7, 23). The term “telenursing” also encompasses a method of remotely providing nursing care or services using devices such as smartphones, computers, or other advanced technologies capable of capturing, saving, processing, sharing, and disseminating information, including text, photographs, and videos through telecommunication (4, 19–21, 24–28). In 1974, Mary Quinn delivered telenursing care to patients at Logan Airport, marking the inception of this practice in the medical industry (7, 20–22).

Innovative technical advancements have brought about significant changes in various fields, impacting both the progress of medicine and the delivery of healthcare services (4, 5, 12, 20, 21). As nurses increasingly enhance and redefine their roles and responsibilities through telehealth nursing, which involves the technology-based application of providing nursing care (16, 18), this emerging field of medical services requires collaboration among clinical professionals, hospitals, health centers, and financial and clinical experts in a digital environment. This collaboration aims to ensure the equitable distribution of medical services, maintain service quality, and enhance the affordability of healthcare (4, 5, 12, 20, 21, 29).

Also, telenursing technologies boost how well patients follow their treatment plans, improve access to nursing care, enhance patient safety at home and other health facilities, and facilitate communication with healthcare providers (4, 12, 19–21). Such an innovative and advanced form of nursing care delivery (telenursing care) allows for the development of new, innovative nursing care models that can enhance nursing healthcare service delivery (1). Since technology influences the quality of nursing care, it is better to include innovative technologies like telenursing, telemedicine, telemonitoring, etc. that enhance and boost the quality of nursing care within the nursing field. Therefore, many research projects can be used to develop, apply, and integrate such types of new technology into nursing practice (1, 2, 7).

Amidst various challenges, the rapid advancement of technology and escalating healthcare costs have prompted the nursing team to leverage technology for enhanced patient care (5, 7, 30). To tackle these challenges, streamline healthcare systems, minimize costs, alleviate the isolation of healthcare personnel, and concurrently enhance patient care outcomes, the healthcare sector is increasingly adopting digital health technologies (5, 7, 31).

Despite growing interest in digital health technologies, there is a notable absence of empirical evidence regarding the attitudes of nurses toward telenursing in resource-limited settings, particularly in Northwest Ethiopia. This gap is significant because understanding the level of telenursing attitudes among nurses plays a crucial role in the adoption and implementation of such technologies. Various studies have indicated that positive attitudes are a key determinant in the successful integration of digital health innovations (32, 33).

However, current literature lacks specific insights into how nurses in resource-limited settings perceive telenursing. Understanding these perceptions is essential for policymakers and planners who aim to implement telenursing effectively within the nursing domain. Without this understanding, efforts to promote telenursing may face unforeseen challenges and resistance. Addressing this research gap is of paramount importance for several reasons:

It provides policymakers and healthcare planners with crucial evidence on nurses' attitudes towards telenursing, which is essential for designing interventions and creating training programs and support systems tailored to nurses’ perceptions and needs. Understanding the factors that influence nurses' attitudes can help in creating a more favorable environment for the adoption of telenursing. Positive attitudes can lead to higher acceptance and more effective utilization of telenursing technologies, ultimately improving patient care and outcomes (32, 33).

In resource-limited settings, efficient allocation of scarce resources is critical Evidence-based insights into nurses' attitudes can guide investments. These investments might include technology, training, and support infrastructure. This ensures that resources are used effectively to support the transition to telenursing. Nurses' attitudes towards telenursing can directly impact the quality of patient care. Positive attitudes are likely to result in more enthusiastic and effective use of telenursing. This leads to better patient monitoring. Communication and overall care delivery especially in remote or underserved areas, are also enhanced by positive attitudes. The evidence demonstrates that fostering positive attitudes towards telenursing among nurses is crucial for successful integration. This is significant for effective delivery. Patient care is particularly improved, It is especially important in resource-limited settings. Underserved settings benefit greatly (4, 34, 35).

Conducting this research in Northwest Ethiopia will contribute to the local evidence base, providing context-specific data that can inform regional and national health policies. This localized evidence is essential for tailoring interventions to the unique challenges and opportunities within the region. In conclusion, exploring and addressing nurses’ attitudes toward telenursing in Northwest Ethiopia is vital for the successful implementation of this technology in resource-limited settings. It will provide the necessary evidence to support informed decision-making, promote the adoption of telenursing, and ultimately enhance the quality of healthcare services in the region.

To realize these benefits, it is crucial to introduce innovative technologies such as telenursing, into the healthcare sector. Therefore, this study aims to address this gap by exploring nurses' attitudes toward telenursing care and associated factors at a specialized teaching referral hospital in the Amhara region of northwest Ethiopia.

## 2 Methods

### 2.1 Study design, area, and period

This research adopted an institutional-based cross-sectional study design, focusing on nurses employed at two specialized teaching referral hospitals situated in the Amhara Region, northwest Ethiopia. The investigation took place over a period spanning from July 28 to December 19, 2022. The University of Gondar Comprehensive Specialized Referral Hospitals and Tibebe Ghion Specialized Teaching Referral Hospitals were chosen as they were the sole teaching and specialized facilities in the Amhara region during the study. These hospitals were selected based on their comparability in terms of personnel composition and the spectrum of services they provided.

### 2.2 Source and study population

The source population for our study encompassed all nurses employed at Tibebe Ghion and the University of Gondar Specialized Teaching referral hospitals, both situated in the Amhara regional state. Meanwhile, the study population specifically consisted of all nurses actively engaged in delivering nursing care within the confines of the University of Gondar and Tibebe Ghion Specialized teaching referral hospitals during the designated data collection period.

### 2.3 Eligibility criteria

Our study included nurses who were employed at the two teaching, specialized referral hospitals and those who willingly volunteered to participate. However, individuals who lacked permanent employment status were not actively working during the data collection period, or faced difficulties reporting to work at the specified time were excluded from participation. This ensured a focus on nurses with consistent employment and availability during the study period.

### 2.4 Determination of sample size and selection procedures

During the study period in the Amhara region, only two specialized teaching referral hospitals, all specialized teaching referral hospitals in the region were considered for inclusion in the study and approached accordingly.

Since there is no evidence or no study was conducted on the telenursing attitude specifically among nursing professionals in the study area we utilized a single population proportion formula to establish the required sample size by considering key factors, such as the standard normal deviation (with *Z a*/2 = 1.96) for a 95% confidence interval (CI), *P*, proportion of telehealth nursing attitude (*P* = 0.5), a margin error (d = 0.005), and the final sample size (*n*). This factor was chosen due to the absence of a previous study in the same area. A precision level (margin of error) of 5% was targeted, resulting in a calculated sample size of 384.

$$n\;=\;\frac{{\left(z_{\frac{a}{2}}\right)}^{2}*pq}{d^{2}}$$

Where *n* represents the required sample size, *d* is the margin of error, the proportion reflecting attitudes toward telenursing, and *q* = 1 − *p*.

$$n\;=\;\frac{{\left(z_{\frac{a}{2}}\right)}^{2}*p(1-P)}{d^{2}}=n\;=\;\frac{{\left(1.96\right)}^{2}*\;0.50(1-0.50)}{{\left(0.05\right)}^{2}}=384$$

To account for a 10% non-response rate, the final total sample was determined to be 423 participants. The selection of study participants involved a two-step process: initially using a straightforward random sampling method and then implementing proportional allocation.

### 2.5 Study variables

This study examined nurses' perspectives on telenursing care, focusing on various factors. The independent variables encompassed demographic aspects like age, gender, job experience, education, and marital status. Additionally, variables included social media/internet use, experience with online patient interactions, internet access at home, preferred platforms, and the source of information. Professional factors such as access to personal computers/desktops, digital training, ICT infrastructure, availability of technical support staff, internet access at the workplace, computer-related training, and nurses' awareness and knowledge of telenursing care were also considered in this research.

### 2.6 Operational definitions

**Attitude toward telenursing care** is an outcome variable that reflects nurses' behavior and thinking, categorized as either poor or good based on previous studies. It was assessed using a 5-point Likert scale with 19 questions, scoring from “1” (strongly disagree) to “5” (strongly agree), resulting in a minimum score of 19 and a maximum of 95. A poor attitude is indicated for participants scoring below the median, while those scoring above the median are considered to have a good attitude, as indicated in prior studies (4, 5, 26, 36–44).

**Awareness of telenursing care** serves as an explanatory variable, representing the extent to which nurses are acquainted with or informed about telenursing care. This awareness was gauged through a set of ten 5-point Likert scale questions, where respondents rated their agreement on a scale from “1” (strongly disagree) to “5” (strongly agree), resulting in individual scores ranging from 10 to 50. Upon a thorough examination of prior studies, these ten questions were utilized to classify nurses into two groups based on their telenursing care awareness. Nurses scoring below the median were identified as having limited awareness, while those scoring above the median were deemed to possess a substantial understanding of telenursing care (4, 7, 26, 38–42).

**Knowledge of telenursing care** is an explanatory variable representing nurses' comprehension of the purpose and advantages of telehealth nursing (telenursing). This understanding is determined by ten questions categorized as indicators of poor or good knowledge. The evaluation involved a set of “yes” or “no” questions, with answers rated as “0 = no” or “1 = yes,” resulting in a score range from “0” to “10”.

Participants were classified as having either poor or strong knowledge based on their median scores. A review of prior studies indicates that participants scoring below the median generally demonstrated poor knowledge of telenursing, while those scoring above the median exhibited a stronger understanding (5, 7, 26, 31, 36–40, 43, 45–49).

### 2.7 Data collection procedures and tools

Data were collected using a pretested, well-structured, self-administered questionnaire. Before developing the data collection tool, a review of relevant literature was conducted (4, 5, 7, 19, 26, 31, 37–49). The questionnaire, designed for self-administration, consisted of a mix of question types. These included a binary “yes” or “no” question, a Likert scale ranging from 1 to 5, and a set of questions categorized into six groups, covering socio-demographic, technological, organizational, awareness, knowledge, and attitude-related aspects.

### 2.8 Data quality management

Before initiating the actual data collection process, both supervisors and data collectors underwent training. Five individuals assigned to data collection from study participants and two supervisors responsible for supportive supervision during data collection received a two-day training session. The training encompassed the study's objectives, relevance, data confidentiality, respondents' rights, informed consent, and data collection techniques.

Overall, our study adapted and verified the questionnaire using a methodical procedure to increase the reliability of our results. First, a review of the existing literature on telenursing and nurses' attitudes towards digital health technologies was done to ensure the items were relevant. Then, expert consultation with healthcare professionals and academic researchers in the field was done to refine the questionnaire and enhance its content validity. Next, the questionnaire was pre-tested on 41 nurses (10% of our total sample size) at Felege Hiwot Specialized Referral Hospital in Bahir Dar City to identify and correct any ambiguities or issues in the questions. Finally, statistical validation methods including Cronbach's alpha were done based on the pre-test results. The results were high, Cronbach's alpha for attitude, knowledge, and awareness questions were 0.946, 0.930, and 0.923 respectively. This thorough adaptation and validation process ensures our results are trustworthy.

### 2.9 Data analysis and processing

We utilized Epi-data version 4.6 for data entry, ensuring daily checks for questionnaire completeness during the data collection period. The collected data underwent coding, and its completeness, absence of missing values, and clarity were verified by both the principal investigator and supervisor at the time of entry.

For analysis, the data was exported to Statistical Package for Social Science (SPSS) version 27. We employed descriptive and inferential statistical analyses, presenting the results through pie charts, bar charts, frequency tables, and percentages. To identify factors influencing nurses' attitudes toward telenursing, we applied a binary logistic regression model.

Correlation strength between dependent and independent variables was measured using adjusted odds ratios with a 95% confidence interval. Initially, a bivariate analysis assessed the significance of each independent variable, considering a cut-off point (*p*-value less than 0.2) to establish relationships (7, 31, 50–53).

Next, a 95% confidence interval and a *p*-value less than 0.05 were used to pinpoint factors significantly associated with nurses' attitudes toward telenursing care. A multi-collinearity test confirmed no significant issues, as all variables showed variance inflation factors (VIF) ranging from 1.047 to 7.611 (7, 31, 54). Finally, model fitness was gauged through the Hosmer and Lemeshow test, with a *p*-value greater than 0.05 indicating statistical significance.

### 2.10 Patients and public involvement

Patients or the public were not involved in the study.

## 3 Results

### 3.1 Socio-demographic characteristics

A set of 423 structured self-administered questionnaires was distributed among nurses at two specialized teaching referral hospitals. The response rate was notable, with 416 completed and returned questionnaires, accounting for 98.345% of the distributed surveys.

Upon analyzing the demographic and personal background data from the participants, it was observed that a substantial portion, specifically 232 individuals (55.8%), belonged to the age group of 23–30 years, with a mean age of 30.0 ± 4.285 years. The academic background of the majority of participants (254, 61.1%) indicated possession of a BSc degree. Additionally, marital status revealed that 198 participants (47.6%) were unmarried, while religious affiliation showed that 204 participants (49.0%) identified as Orthodox Christians (Table 1).

**Table 1:** Shows the sociodemographic details of the nurses in Northwest Ethiopia in 2022–2023 (*n* = 416).

Socio demography factors	Attribute	Frequency	Percent
Age in a year	23–30 Year	232	55.8%
	above 30 Year	184	44.2%
Sex	Male	192	46.2%
	Female	224	53.8%
Educational statues	Diploma	79	19.0%
	Degree	254	61.1%
	Master	83	20.0%
Marital status	Married	175	42.1%
	Unmarried	198	47.6%
	Divorced	23	5.5%
	Windowed	20	4.8%
Religion	Orthodox	204	49.0%
	Muslim	104	25.0%
	Protestant	59	14.2%
	Catholic	29	7.0%
	Josh	20	4.8%
Monthly salary in ETB	4,609–6,193 ETB	187	45.0%
	6,194–9,056 ETB	151	36.3%
	Above 9,056	78	18.8%
Working experience in a year	0–3 Year	195	46.9%
	4–7 Year	132	31.7%
	Above 7 Year	89	21.4%

### 3.2 Technological related factors

Among study participants, 295 (70.9%) had personal computers, and the majority of them (75.0%) used social media, with 64.6% and 35.7% of them usually using Facebook and Telegram, respectively (see Table 2).

**Table 2:** Technological factors influencing telenursing attitudes Among nurses in North-west Ethiopia (2022/23) (*n* = 416).

Technological factors	Response	Frequency	Percent
Personal Computer Utilization	No	295	70.9
	Yes	121	29.1
Internet Use OR Social Media Use	No	104	25.0
	Yes	312	75.0
Social Media Usage Patterns	Facebook	242	58.2
	Telegram	148	35.6
	Twitter	86	20.7
	Instagram	83	20.0
	Email	81	19.5
Interact with patients through social media using the internet at home	No	291	70.0
	Yes	125	30.0
	No	285	68.5
	Yes	131	31.5

### 3.3 Study participants' sources of information

**Figure 1:**
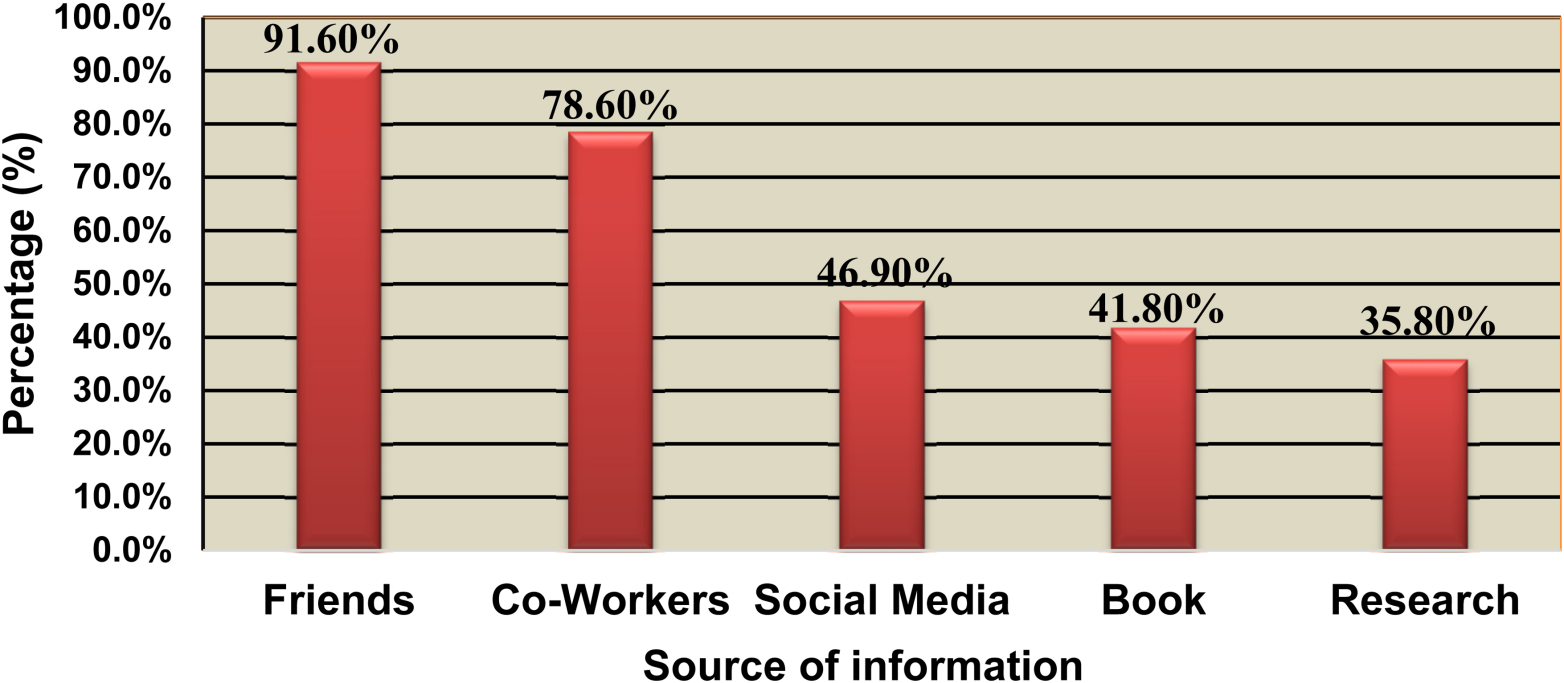
The major source of information for nurses in Northeast Ethiopia in 2022/23 (*n* = 416).

The primary sources of information for the majority of study participants were friends, accounting for about 91.6%, and coworkers, accounting for 78.6% (see Figure 1).

### 3.4 Organizational related factors

Out of the total study participants, 193 (46.4%) underwent computer training, 190 (45.7%) nurses had desktop access in their workplace or organizations, and 334 (80.3%) and 324 (77.9%) participants reported the availability of ICT infrastructure and technical support in their organization, respectively (see Table 3).

**Table 3:** Organizational factors influencing telenursing attitudes Among nurses in North-west Ethiopia, 2022/23 (*n* = 416).

Organizational factors	Response	Frequency	Percent (%)
Access to desktop computers at work	No	226	54.3
	Yes	190	45.7
Organizations facilitate online instruction	No	245	58.9
	Yes	171	41.1
The organization's ICT infrastructure	No	82	19.7
	Yes	334	80.3
ICT Technical Support staff in your organization	No	92	22.1
	Yes	324	77.9
Access to the Internet within the organization	No	223	53.6
	Yes	193	46.4
Computer Training in the organization	No	223	53.6
	Yes	193	46.4

### 3.5 Level of telenursing care awareness, knowledge, and attitude among nurses

**Figure 2:**
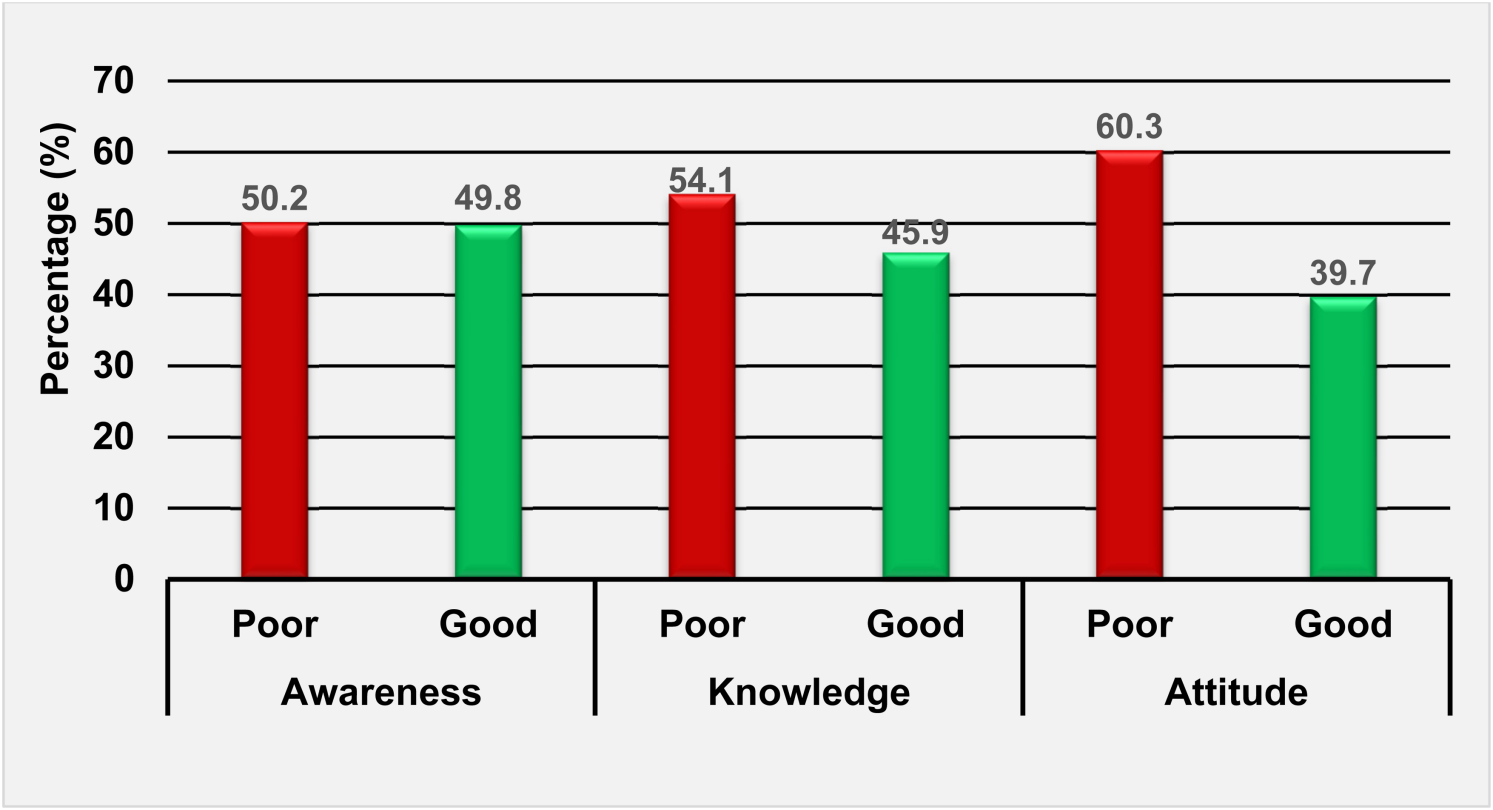
Nurses’ awareness, knowledge, and attitude toward telenursing care in North-west Ethiopia, 2022/23 (*n* = 416).

In this study, 49.8%, 45.9%, and 39.7% of nurses demonstrated good awareness, knowledge, and attitudes toward telenursing care, respectively (see Figure 2).

### 3.6 Analysis of binary logistic regression to identify variables related to nurses' attitudes toward telenursing care

In the bivariable analysis, nurses' attitudes toward telenursing care demonstrated significant associations (*p* < 0.2) with various factors, including awareness, knowledge, age, sex, educational level, job experience, monthly salary, social media/internet use, personal computer access, preferred social media platforms, experience of online patient contact, source of information, internet access at home, digital training, desktop computer access at workplaces, internet access in organizations, and computer-related training.

Upon examining the final multivariable logistic regression model, it was observed that several variables continued to exhibit a statistically significant association with nurses' attitudes toward telenursing care. These variables included awareness (AOR = 4.24; 95% CI: 1.99–9.041), knowledge (AOR = 2.45; 95% CI: 1.10–5.46), job experience (4–7 years: AOR = 2.74; 95% CI: 1.72–4.37, and above 7 years: AOR = 3.46; 95% CI: 2.05–5.85), personal computer access (AOR = 2.66; 95% CI: 1.26–5.63), social media and internet use (AOR = 6.24; 95% CI: 2.06–18.92), organizational computer access (AOR = 2.25; 95% CI: 1.12–4.51), digital training (AOR = 3.32; 95% CI: 1.65–6.66), internet access in the organization (AOR = 6.10; 95% CI: 3.02–12.33), and computer-related training (AOR = 4.01; 95% CI: 2.06–7.78) (Table 4).

**Table 4:** Regression analysis, both bivariable and multivariable, of the variables linked to nurses’ attitudes about telenursing care in Northwest Ethiopia 2022/23 (*n* = 416).

Variable	Category/responses		Level of telenursing attitude		OR (95%, CI)	
			Good No (%)	Poor No (%)	COR (95% CI)	AOR (95% CI)
Awareness	Poor		28 (13.4)	181 (86.6)	1	1
	Good		137 (66.2)	70 (33.8)	12.65 (7.74–20.68)	4.24 (1.99–9.041)**
Knowledge	Poor		37 (16.4)	188 (83.6)	1	1
	Good		128 (67.0)	63 (33.0)	10.32 (6.49–16.42)	2.45 (1.10–5.46)*
Educational level	Diploma		23 (29.1)	56 (70.9)	1	1
	BSc. Degree		97 (38.2)	157 (61.8)	1.50 (0.87–2.60)	1.52 (0.48–4.84)
	Master		45 (54.2)	38 (45.8)	2.88 (1.51–5.52)	2.57 (0.15–45.30)
Job work experience in the year	0–3 Year		51 (26.2)	144 (73.8)	1	1
	4–7 Year		65 (49.2)	67 (50.8)	2.74 (1.72–4.37)**	2.28 (0.5–9.31)
	Above 7 Year		49 (55.1)	40 (44.9)	3.46 (2.05–5.85)**	2.57 (0.43–15.48)
PC/Laptop computer access	No		91 (30.8)	204 (69.2)	1	1
	Yes		74 (61.2)	47 (38.8)	3.53 (2.27–5.49)**	2.66 (1.26–5.63)*
Social media/Internet use	No		21 (20.2)	83 (79.8)	1	1
	Yes		144 (46.2)	168 (53.8)	3.39 (2.0–5.74)**	6.24 (2.06–18.92)**
Social media usually used	Facebook	No	52 (29.9)	122 (70.1)	1	1
		Yes	113 (46.7)	129 (53.3)	2.06 (1.36–3.10)**	1.39 (0.49–3.90)
	Telegram	No	92 (34.3)	176 (65.7)	1	1
		Yes	73 (49.3)	75 (50.7)	1.86 (1.24–2.80)**	0.51 (0.21–1.25)
	Twitter	No	120 (36.4)	210 (63.6)	1	1
		Yes	45 (52.3)	41 (47.7)	1.92 (1.19–3.10)**	1.32 (0.44–3.93)
	Instagram	No	118 (35.4)	215 (64.6)	1	1
		Yes	47 (56.6)	36 (43.4)	2.38 (1.46–3.88)**	1.81 (0.58–5.69)
	Email	No	126 (37.6)	209 (62.4)	1	1
		Yes	39 (48.1)	42 (51.9)	1.54 (0.945–2.51)	0.33 (0.10–1.08)
An experience in contacting online		No	102 (35.1)	189 (64.9)	1	1
		Yes	63 (50.4)	62 (49.6)	1.88 (1.23–2.88)**	0.61 (0.29–1.29)
Internet access at home		No	99 (34.7)	186 (65.3)	1	1
		Yes	66 (50.4)	65 (49.6)	1.91 (1.25–2.91)**	0.53 (0.25–1.13)
Source of Information	Friends	No	9 (25.7)	26 (74.3)	1	1
		Yes	156 (40.9)	225 (59.1)	2.0 (0.91–4.39)	1.50 (0.48–4.68)
	Social Media	No	70 (31.7)	151 (68.3)	1	1
		Yes	95 (48.7)	100 (51.3)	2.05 (1.38–3.05)**	0.91 (0.40–2.08)
	Research Articles	No	70 (26.2)	197 (73.8)	1	1
		Yes	95 (63.8)	54 (36.2)	4.95 (3.22–7.62)**	1.87 (0.85–4.09)
Desktop computer access at the workplace		No	46 (20.4)	180 (79.6)	1	1
		Yes	119 (62.6)	71 (37.4)	6.56 (4.24–10.16)**	2.25 (1.12–4.51)*
Organizations facilitate digital training		No	53 (21.6)	192 (78.4)	1	1
		Yes	112 (65.5)	59 (34.5)	6.88 (4.44–10.66)**	3.32 (1.65–6.66)**
Internet access in the organization		No	39 (17.5)	184 (82.5)	1	1
		Yes	126 (65.3)	67 (34.7)	8.87 (5.63–13.99)**	6.10 (3.02–12.33)**
Computer Training in the organization		No	56 (25.1)	167 (74.9)	1	1
		Yes	109 (56.5)	84 (43.5)	3.87 (2.55–5.86)**	4.01 (2.06–7.78)**

*p-value < 0.05; **p-value < 0.01.

## 4 Discussion

This study investigates nurses' attitudes toward telenursing care and the factors influencing these attitudes in northwest Ethiopia. The results reveal that 39.7% (95% CI: 35.1%-44.5%) held a positive view of remote nursing assistance (telenursing). This aligns with a similar study in India, where 61% and 39% demonstrated low and high attitudes, respectively, toward telehealth (26).

In contrast, our findings differ from research in northwestern Ethiopia, where 64% of healthcare professionals favored telemedicine (5). This discrepancy may be attributed to variations in the included health professionals, the study's scope covering three hospitals, and differences in sample sizes.

Previous investigations in Egypt and Iran reported diverse attitudes toward telemedicine among healthcare professionals. In Egypt, 24.6% and 75.4% of dermatologists had poor and good attitudes, respectively (44). In Iran, about 36% and 63% of health professionals expressed unfavorable and favorable attitudes, respectively, toward telemedicine at Isfahan University of Medical Science (38). Another study in Iran found that a significant 73% of healthcare workers had a positive attitude toward telemedicine (41, 42). Similarly, a study in Poland found that 71% of university nursing students had a favorable view of telehealth and telenursing (4).

These variations may stem from differences in technology utilization, internet access, personal computer availability, and institutional computer accessibility. Notably, only 61.2% of our study participants had personal computers, and 50.4% had internet access at home, contrasting sharply with the study in Iran where 99.2% could use a computer and access the internet (41, 42).

Among participants with a positive attitude toward telenursing care, 46.2% used social media/internet in their daily activities, while 48.7% sourced information from social media. This is consistent with a study in Iran where 52.6% of participants obtained information from public media and the Internet (41, 42). In our study, 33.7% of nurses agreed to use telenursing in the future, slightly higher than the study in Iran where only 30.4% were willing. Additionally, 36.7% of nurses in our study acknowledged that utilizing telenursing could enhance the efficiency of nursing staff, aligning with a similar study in Iran (4, 19).

Approximately 30% of the respondents in our study believe that telenursing may enhance nursing care services, a perspective contrasting with an Australian study where 52% agreed that e-health can improve nursing practices (47). However, only 39.4% of the nurses in our study agreed that telenursing may enhance nursing care quality, a figure significantly lower than the Polish study where over 70% believed in its utility across medical specialties (4, 19). This difference may arise from variations in educational systems, technology use, and internet access across countries. In contrast, 44.2% and 31.1% of the participants in our study agree that telenursing may improve nursing communication and decrease unnecessary travel costs, respectively, aligning with findings from research conducted in Ethiopia (5) and India (26).

About 29.3% of participants agree or strongly agree that telenursing improves nursing clinical decision-making, while 24.7% believe it may threaten information privacy. This finding contrasts with a study in North West Ethiopia, where 54.0% and 66% expressed agree and strongly agree on the positive impact of telemedicine on clinical decision-making quality and its potential threat to information confidentiality (5).

Approximately 73.6% of nurses in our study indicate that telenursing technology demands more effort, consistent with a study in North West Ethiopia, where 72.6% believed that Telemedicine technology required increased effort (5).

Similarly, a study in Iran found that 76.2% of participants perceived telemedicine as complex, requiring substantial mental effort (4).

The results indicate that nurses' awareness (AOR = 4.24, 95% CI: 1.99–9.041) and knowledge (AOR = 2.45, 95% CI: 1.10–5.46) were significantly associated with positive attitudes toward telenursing care. This aligns with previous studies that have highlighted the importance of awareness and knowledge in shaping healthcare professionals' perceptions and acceptance of telemedicine technologies (55, 56). This might be due to increasing nurses' awareness and knowledge of telenursing and its capabilities, benefits, and applications in healthcare increasing their perception of telenursing technology for use in nursing clinical care.

The findings suggest that nurses with 4–7 years (AOR = 2.74, 95% CI: 1.72–4.37) and over 7 years (AOR = 3.46, 95% CI: 2.05–5.85) of job experience were more likely to have positive attitudes toward telenursing. This could be attributed to experienced nurses' familiarity with technological innovations and their ability to recognize the potential benefits of telenursing in resource-limited settings (57, 58). Potential Reasons for the Findings are Experienced nurses are more familiar with technological innovations in healthcare. As they have worked in the field for longer, they have likely seen the introduction and adoption of various new technologies. This familiarity may make them more open to and appreciative of the potential of telenursing, experienced nurses can better recognize the benefits of telenursing, especially in resource-limited settings. With their extensive experience, they understand the challenges of providing quality care with limited resources. They may see telenursing as a way to expand access to care and improve outcomes in these settings, and more experienced nurses may be more comfortable with the autonomy and independence that telenursing can provide. As they progress in their careers, they may value the ability to work remotely and have more control over their schedules.

The study revealed that personal computer access (AOR = 2.66, 95% CI: 1.26–5.63), social media and internet use (AOR = 6.24, 95% CI: 2.06–18.92), organizational computer access (AOR = 2.25, 95% CI: 1.12–4.51), and internet access in the organization (AOR = 6.10, 95% CI: 3.02–12.33) were all significantly associated with positive attitudes toward telenursing. These findings are consistent with previous research, which has highlighted the importance of technological infrastructure and access in the successful implementation of telemedicine (55, 56). By addressing these technological barriers and providing nurses with the necessary tools and support, healthcare organizations can create an environment that is conducive to the acceptance and implementation of telenursing services.

The study found that digital training (AOR = 3.32, 95% CI: 1.65–6.66) and computer-related training (AOR = 4.01, 95% CI: 2.06–7.78) were also significantly associated with positive attitudes toward telenursing. This aligns with existing literature, which suggests that providing targeted training and skill development opportunities can enhance healthcare professionals' confidence and willingness to engage with telemedicine technologies (56, 58). The potential reason for this fact is that comprehensive training programs for healthcare professionals facilitate the successful implementation and adoption of telenursing technologies. By equipping nurses and other healthcare providers with essential digital and computer-related skills, organizations can create a more positive and receptive environment for integrating telenursing into clinical practice.

## 5 Strengths and limitations of the study

This research examines the current attitudes of nurses toward telenursing care in Ethiopia. The findings represent the first exploration of nurses' perspectives on telenursing care within the country's nursing practice. The significance of this research extends to policymakers, program developers, and project planners in the nursing sector, providing a foundational reference for future researchers with similar interests. However, the study has limitations. It does not extensively explore the challenges and barriers to the implementation of telenursing in resource-limited settings. Additionally, the study is an institution-based cross-sectional survey conducted exclusively in two dedicated educational referral hospitals within the Amhara region, thereby excluding other referral hospitals. Consequently, the results are only applicable to these specific institutions.

## 6 Conclusion

The results of this survey indicate that a significant portion of nurses hold a negative opinion regarding telenursing services. This can be attributed to their limited awareness, insufficient knowledge, and a lack of training in computer and digital health-related aspects. The utilization of technology in nursing practice involves various considerations, such as the ability to engage in online communication and the accessibility of computers and the Internet.

Moreover, a nurse's attitude towards telenursing care is intricately connected to factors such as education level, knowledge, access to computers and the internet, and training in digital health. In light of these findings, it is imperative for key stakeholders, including Federal Ministers of Health (FMoH), institutions of higher education such as universities or colleges, and both governmental and non-governmental organizations engaged in nursing care, to collaborate. This collaborative effort aims to enhance nursing practices by addressing and improving nurses' attitudes toward the acceptance, utilization, and implementation of advanced technology in the nursing domain.

## Data Availability

The data analyzed in this study is subject to the following licenses/restrictions: the data generated during the course of this study will be made available upon reasonable request from the corresponding author. However, due to the nature of the study area being one of the surveillance sites in the country, we are unable to publicly release the data. Access to the data is restricted by the University of Gondar, College of Medicine and Health Sciences, which owns the data. Therefore, interested parties can request access to the data and supplementary information by contacting the surveillance site coordinator office at the University of Gondar, College of Medicine and Health Sciences via email. Requests to access these datasets should be directed to kassahuna@yahoo.com.
